# Qatar’s assisted home hemodialysis program: A beacon of hope for the vulnerable patient

**DOI:** 10.5339/qmj.2024.28

**Published:** 2024-07-31

**Authors:** Abdullah Hamad, Mohamed Yahya Abdelhai, Mostafa Elsherbiny, Ahamad Abdelwahed, Hoda Tolba, Sandhya Chembolu, Fadumo Yasin, Rania Ibrahim, Shajahan Joseph, Teha Almuhanadi, Mohamad Alkadi, Hassan Al Malki

**Affiliations:** 1 Department of Medicine, Division of Nephrology, Hamad Medical Corporation, Doha, Qatar *Email: ahamad9@hamad.qa; 2 Metcocare Company, Doha, Qatar; 3 D.med Healthcare, Hyderabad, India

**Keywords:** Dialysis center, travel distance, time, spatial, accessibility

## Abstract

In a bold departure from conventional healthcare paradigms, Qatar’s Assisted Home Hemodialysis (AHHD) national program stands as a testament to Hamad Medical Corporation’s unwavering commitment to excellence. This innovative care model is tailored to address the distinct challenges of hemodialysis patients, particularly the elderly, who require ambulance transport. It provides them with the convenience of at-home dialysis with the full support of dedicated nursing care. AHHD included 76 patients from July 2021 to December 2022. It has significantly improved patients’ quality of life, achieving an exceptional 99% satisfaction rate with an extremely low complication rate. It has also delivered tangible improvements in health outcomes, marked by a reduction in hospitalizations, decreased transmission of COVID-19, cost-effectiveness, alleviating strain on ambulance services, and reducing demand for dialysis slots and manpower. In conclusion, the clinical and financial success of the AHHD program positions it as a superior alternative to traditional in-center dialysis, particularly in its capacity to cater to the needs of the most complex and challenging patient populations.

## Introduction

End-stage kidney disease (ESKD) prevalence has increased significantly in the last few decades, primarily due to improvements in patients’ survival, reaching more than 2.6 million people on dialysis worldwide, and numbers are expected to double by 2030.^1,2^ Dialysis-dependent patients can be treated either in an ambulatory clinic [in-center hemodialysis (HD)] or at home [home hemodialysis (HHD) or peritoneal dialysis (PD)].^3^ Although dialysis started in the 1960s primarily as HHD, over the past several decades, it has shifted mainly to in-center HD.^4^

The cost of dialysis care for ESKD patients is enormous and carries a high burden on healthcare resources, sometimes exceeding 90.6 thousand US dollars per patient annually.^5^ Overall, Medicare expenditures related to providing care for patients with ESKD totaled more than 35.9 billion US dollars in 2017, translating to ~7.2% of total Medicare-paid claims.^5^ Currently, most HD patients receive treatment in the form of in-center HD, despite some promising evidence of cost-savings by using home-based therapies.^6-8^ Therefore, cost-effective therapies such as home-based dialysis should be considered.

HHD is usually done as self-care by the patients themselves through a portable hemodialysis machine under training and monitoring by a dialysis team. It offers greater patient autonomy, cost benefits, and treatment-related flexibility, and its outcomes showed improved quality of life and patient survival compared to traditional in-center HD.^9-15^ The FHN trial16 showed favorable outcomes with composite outcomes of death or change in left ventricular mass and death or change in a physical-health composite score compared to traditional HD. Despite its advantages, the overall uptake of HHD remains low, ranging between 0.41% in developed Asian countries, 2.1% in the United Kingdom, and 9.4% in Australia.^17,18^ The explanation may be due to concerns about housing constraints, social isolation, apprehension of self-care, and family burdens.^18,19^ During the coronavirus disease 2019 (COVID-19) pandemic, the design of many healthcare facilities has been changed to minimize the risk of infection transmission. The fact that dialysis patients are at a higher risk of developing a severe COVID-19 infection compared to the general population20 should emphasize the importance of shifting a considerable number of patients to perform dialysis at home. Many recent data demonstrated that in-center HD patients are up to three times more susceptible to developing COVID-19 infection compared to HHD patients,^21,22^ and hospitalization due to COVID-19 infection in in-center HD patients was 3–4 times that of HHD or PD patients.^23^ These findings have prompted urgency among nephrologists to switch more patients to these home dialysis programs.

Considerable numbers of dialysis patients may not be able or willing to commit to home-based dialysis therapy due to cognitive and physical barriers, as well as a lack of support from family and community.^24-27^ The Assisted Home Hemodialysis (AHHD) program was designed to decrease the burden older patients and their caregivers may face at home. The assisted model has been used successfully in assisted home PD therapy in well-established programs in some countries, and the results demonstrated acceptable quality of life and equivalent clinical outcomes in terms of peritonitis, technique survival, and hospital readmissions.^28-31^ AHHD is a new concept that allows the dialysis team to provide HD at home. Usually, it is done by a visiting dialysis nurse using a mobile HD machine. On the contrary, assisted home HD programs are very uncommon due to financial and logistical restrictions.

Very little evidence exists on the utilization of such programs except in specific environments (nursing homes and special patient cases).^32,33^

## Dialysis Services in Qatar

**Figure 1.**
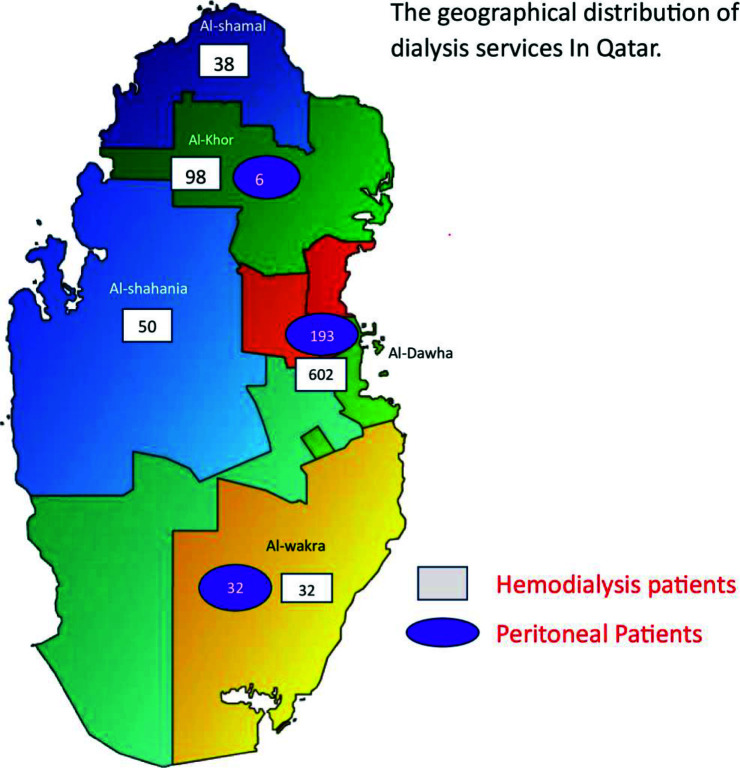
Geographical distribution of dialysis service in the State of Qatar.

ESKD prevalence is increasing in Qatar and is expected to go up by 30% by the end of 2030.^34^ Dialysis services in Qatar are solely provided by Hamad Medical Corporation (HMC). HD, PD, and AHHD are available and offered to patients with ESKD.^35,36^ There are seven HD centers distributed across different areas in Qatar (Figure 1). The PD program is large and well-established, serving over one-fifth of the ESKD population.^37^ AHHD was initially started in 2011, but it was approved only to serve a very few selected cases of elderly, bedridden patients with multiple comorbidities requiring ambulance transportation to dialysis centers. Although AHHD was convenient for such patients, it was labor-intensive and could not be expanded despite reported good outcomes and high demand, with more bed ridden patients requiring ambulance transportation due to limited resources and budget cut back.

## Pre-AHHD Landscape: Understanding the Situation Before Implementation

Over half of our dialysis patients are elderly, and many of them have limited mobility, necessitating a wheelchair or stretcher to reach the dialysis center.

More than half of these mobility-restricted individuals rely on ambulance services for transportation to dialysis centers three times a week, exerting substantial pressure on our local ambulance services. It also leads to traffic during peak hours at the dialysis center for loading and offloading. The reliance on ambulances for transportation not only compromises the dignity and comfort of these elderly patients but also inflicts psychological distress, particularly for those with dementia or cognitive impairments. Prolonged wait times for ambulance services following dialysis sessions further disrupt their treatment schedules and impact the care of other patients needing timely dialysis.

The long-distance travel and the three-weekly dialysis regimen may lead to burnout and missed treatment sessions. The frequent transportation of elderly dialysis patients with restricted mobility with constant movement between beds and stretchers or wheelchairs increased the risk of falls and fractures due to their mineral and bone disease related to age, risk factors, and ESKD. Moreover, strict regulations limited family members’ presence, further isolating them (especially during COVID-19 times). These complications often led to repeated hospitalizations and increased medical expenses.

The concept changes from in-center dialysis to home hemodialysis met with some resistance and fear from patients and family members. This resistance was overcome by providing awareness, along with proof from evidence-based practices across the globe, illuminating the safety and efficacy of HHD.

Considering these challenges, initiating an AHHD program emerges as a potential solution to enhance the overall well-being and care quality of this vulnerable elderly population. Addressing specialized transportation, cost-efficiency, personnel training, reduced wait times, improved patient comfort, and alternative care options can significantly improve the care experience by providing dialysis treatment in the comfort of their homes, vastly enhancing the care experience for elderly dialysis patients.

## Beyond Limits: AHHD’s Goals for Holistic Progress

This AHHD initiative, sponsored by the Ministry of Health and Hamad Medical Corporation, is specifically designed to address the quality-of-life needs and pressing concerns of the elderly dialysis population in Qatar, who rely primarily on ambulance transportation. This approach aims to reduce hospitalizations, medical costs, strain on ambulance services and dialysis capacity, and improve overall care.

Our manuscript objective is to describe the initiation and implementation of assisted home hemodialysis in Qatar and to describe the impact on improving the quality of life, decreasing ambulance services, and reducing medical costs among the elderly dialysis population in Qatar.

## The AHHD Project Implementation Journey

This is a qualitative descriptive study of adult hemodialysis patients at all ambulatory dialysis centers in Qatar who were shifted from in-center hemodialysis to assisted home hemodialysis between July 2021 and December 2022.

All case data were obtained from Cerner, including demographic data (age, gender, dialysis treatment duration), comorbidities, dialysis adequacy, vascular access types (arteriovenous fistula, arteriovenous graft, and permanent catheter), vascular access complications (catheter malfunction, infection, and bleeding), hospitalization, incidence of falls, technical incidents episodes, and need for transportation.

A quality of life and satisfaction survey were done per our quality department standard questionnaire per our dialysis routine. It includes questions regarding cost analyses done based on available estimated current cost of our in-center HD (plus ambulance cost) versus the actual cost of the new service in AHHD.

### A- The journey

A compelling narrative of unity and cooperation unfolded in Qatar with the inception of the AHHD program, which saw significant expansion in July 2020. This initiative seamlessly brought together a diverse group of experts, each contributing their own distinct skills to the collective mission. For over a year, the committed team from all collaborating parties worked diligently, meticulously crafting clinical guidelines and policies, fostering collaborative bridges, and addressing every concern to ensure the program’s triumph. Rigorous planning and coordination ensued with partners from Metcocare and D.med Healthcare, further refining processes and polishing guidelines in collaboration with HMC. As the program transitioned into its implementation stage, additional efforts were made to provide education to health care providers, gain support, and deliver home health care services to eligible patients. The culmination of these efforts materialized in July 2021, with the inaugural first AHHD session under the new collaboration, marking a momentous occasion. Over the subsequent 18 months, progress unfolded gradually but consistently, surmounting various challenges. Seventy-six patients actively participated in the program, each contributing their own inspiring narratives. The multidisciplinary team, dedicated to the well-being of these patients, expanded to meet their evolving needs. This sequence provides a summarized account of the remarkable journey undertaken by these patients.

**Figure 2.**
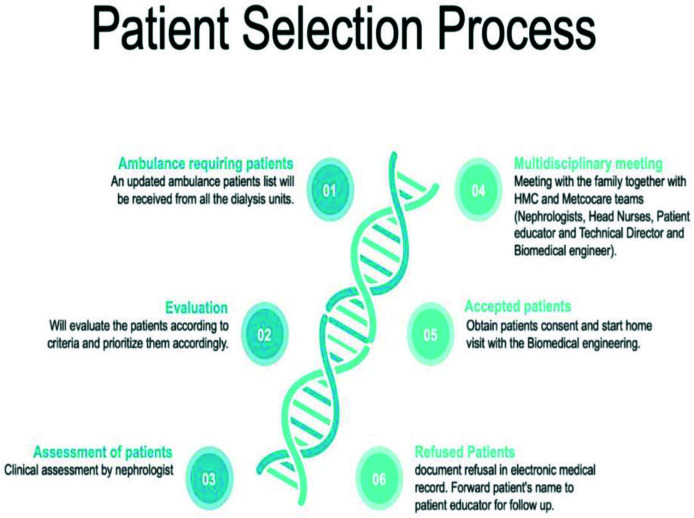
Patient selection process for the Assisted Home Hemodialysis Program.

A- Patient recruitment for the program involves a meticulous process. Identification is based on specific inclusion criteria, focusing on elderly HD patients with multiple comorbidities and restricted mobility, primarily reliant on ambulance transportation. Prior to enrollment, a thorough physician assessment is conducted to ensure a stable medical condition for the home environment (no severe hypotension, sepsis, psychiatric, or behavioral issues that can affect assisted home therapy). Following this, patients and their families are provided with comprehensive education about the AHHD program, allowing them to make informed decisions regarding participation. Subsequently, a detailed multidisciplinary meeting is organized with the patient and/or their family, outlining rights, duties, and expectations from the program. A checklist containing essential program facts is discussed and agreed upon before formal enrollment. Figure 2 briefly illustrates the selection criteria and the preparation algorithm for initiating patients on AHHD.

**Figure 3.**
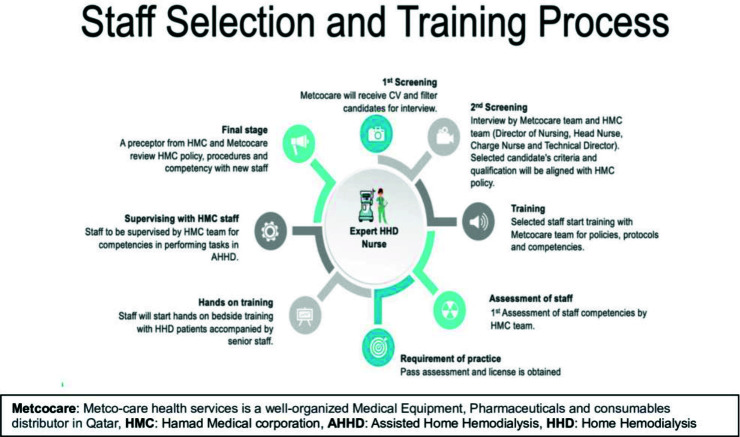
Staff selection and training process for the assisted home hemodialysis program.

B- The selection and recruitment of staff for this unique program involved the development of a comprehensive process aimed at building a highly qualified team. A prerequisite for potential candidates was a minimum of 3 years of experience in dialysis care. The evaluation began with an initial review of the candidates’ credentials, followed by a thorough interview process. Successful candidates who met all the necessary requirements proceeded to an in-depth theoretical training phase. This phase encompassed a detailed review of dialysis fundamentals and a comprehensive understanding of all relevant HMC guidelines, protocols, and policies. Subsequently, candidates undertook practical training tailored specifically to the program’s requirements. This meticulous approach ensures that the selected staff not only have the essential experience but are also knowledgeable in the specific protocols and guidelines essential for the program’s success (Figure 3).

**Figure 4.**
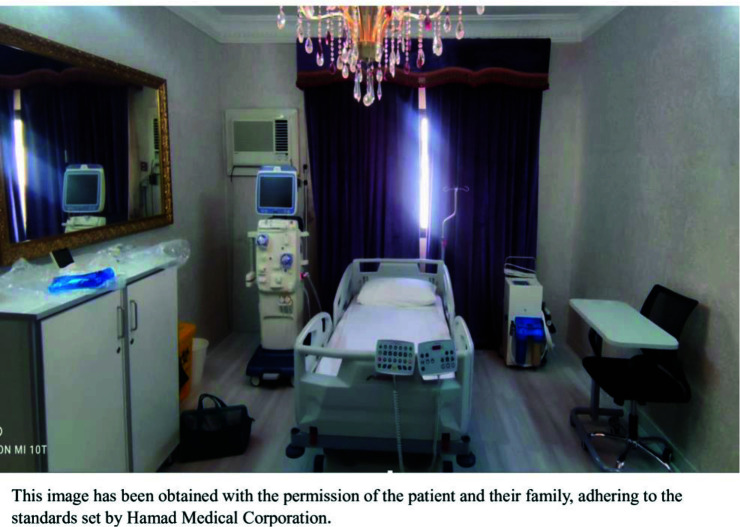
Image describing the interior of patient room participating in the Assisted Home Hemodialysis Program.

C- House inspection and installation: A team from dialysis nursing, dialysis technologists, and a biomedical engineer from HMC would visit the patient’s house. The room would be inspected for space, electricity, and water connection. Suggestions would be proposed for the installation of water connections for the dialysis machine and portable reverse osmosis (which would be stationed in the room). A small closet with a lock is provided to store dialysis consumables. A small additional water tank and water chiller (due to the hot weather in the area) to supply water exclusively for dialysis need to be installed. Water quality would be tested by colony formation unit (CFU) (should be < 100 CFU/ml) and endotoxin level (should be < 0.25 EU/ml). Before each dialysis session, the water will be tested for chlorine, chloramine, hardness, and pH (Figure 4).

**Figure 5.**
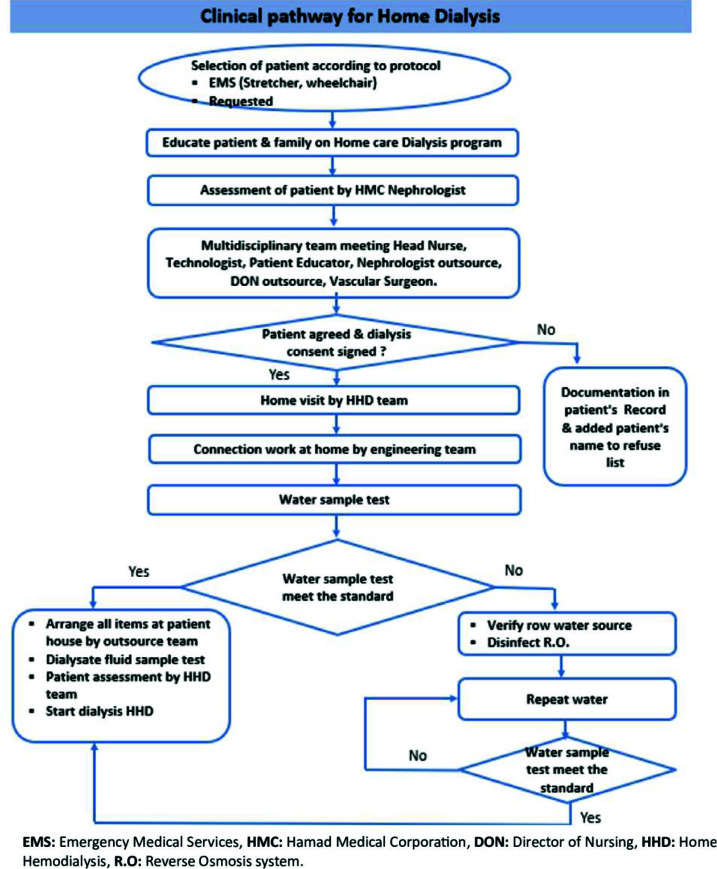
Clinical pathway for Assisted Home Hemodialysis Care.

D- The initiation of AHHD therapy involves a carefully phased approach to ensure a seamless transition for patients adopting this new treatment modality. Initially, the process begins with a patient undergoing dialysis under the direct supervision of the healthcare team. During these initial 2–3 sessions, the HMC team is present to not only provide reassurance to the patient but also to oversee and ensure that the healthcare staff follows all optimal steps in conducting dialysis. This hands-on involvement aims to instill confidence in patients, address any concerns they may have about the new therapy, and guarantee a smooth and successful integration of assisted home HD into their healthcare routine (Figure 5).

E- Effective monitoring of the AHHD program is essential for its success. A rigorous approach was adopted, employing standard key performance indicators (KPIs) to meticulously track various aspects. The HMC team conducts regular meetings to examine the program’s progress, address patient concerns, and escalate issues to a multidisciplinary level when necessary. Comprehensive monitoring encompasses key metrics such as mortality rates, hospitalization, missed treatments, patient retention, and various types of treatment failures, including technical and vascular access issues. This proactive monitoring strategy aims to ensure the program’s optimal performance and the delivery of high-quality care to patients.

### B- Breaking barriers: understanding and addressing AHHD challenges

Several obstacles had to be overcome during the initiation of the AHHD program.

Challenges related to patients and families, setting up the program, training, technical, consumable, transition of patients, COVID-19 pandemic, and medications were faced during initiating the program. Frequent meetings were conducted (once or twice weekly), especially in the program’s first year, to address these challenges. A multidisciplinary approach, meticulous planning, administration support, robust communication strategies, education, and trying to find innovative solutions led to the resolution of most challenges. Table 1 summarizes the challenges met in establishing our assisted home hemodialysis program.

### C- AHHD program: positive impact for all stakeholders

1. Patients: Besides improving quality of life, the program has significantly improved treatment adherence by transitioning from an in-center to an AHHD program, which almost eliminated missed dialysis sessions, resulting in uninterrupted treatment and better health outcomes.

2. Family perspective: The significant convenience to families cannot be overstated, offering physical relief and mental peace by dramatically reshaping the dialysis experience into a more humane and personalized one. It provides care in the comfort of patients’ homes, eliminating the need for families to adjust their schedule around in-center visits, significantly decreasing noncompliance with in-center treatments by removing transportation barriers, and ensuring patients receive consistent care. Patients can experience a more comfortable and convenient treatment process by eliminating the stress and discomfort of hospital visits.

3. Healthcare staff: HMC resources are now being utilized to their fullest potential, with relief of pressure on ambulance services required for dialysis patients’ transportation being a prime example. They reduced hospital-based dialysis sessions. They overall led to improved utilization efficiency of staff in general.

4. Financial stakeholders: The program has resulted in significant cost savings. This cost-effective approach benefits both the healthcare system and patients.

In conclusion, the AHHD Program provides better patient treatment outcomes, less stress for families, optimized resources for staff, and substantial cost savings for financial stakeholders.

## From Progress to Results: AHHD’s Impact

**Figure 6.**
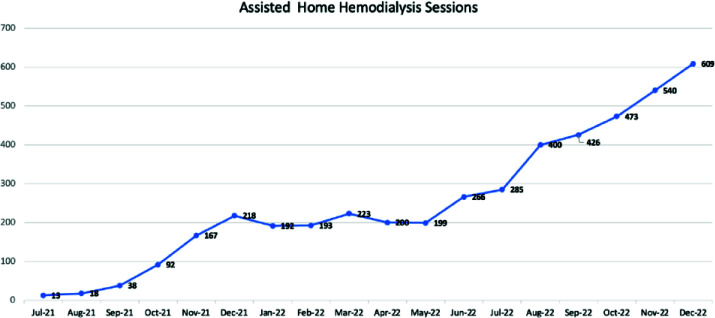
Accumulating number of AHHD sessions in 18 months period.

All patients on ambulatory dialysis service were screened for the program between July 2021 and December 2022. About 50% of eligible patients declined to participate, primarily for feeling safer in the dialysis center setting or having an improper home environment. Seventy-six patients accepted and started AHHD. The mean age was 73 ± 11 years (> 70% of patients were > 70 years old), with 32 males (42%) and 44 females (58%). The mean follow-up period was 7 ± 4 months and accumulated over 4000 dialysis treatments (Figure 6). Twelve patients died, and two patients returned to the dialysis center during the follow-up period (both wanted to socialize with other patients in the center). Deaths were mostly related to infectious or cardiovascular disease causes. No deaths occurred during dialysis or were related to ESKD or dialysis (like hypotension, hyperkalemia, or volume overload). Dialysis adequacy in target (Kt/V > 1.2) was achieved in > 90% of patients. We had 55 patients with permcath, which constituted 72.3% (55 patients) versus 27.7% with arteriovenous fistula (21 patients). Eight incidents of dialysis catheter malfunction occurred during follow-up. Six incidents were resolved with tPA installation in the house setting, and only two required catheter exchange (one had a catheter-related infection). No significant access bleeding, falls, or hypotension episodes were reported. During the study, we had 20 technical incidents related to electricity or water supply failures. All incidents were resolved without significant interruption of dialysis treatments. COVID-19 infection was only 5.5% compared to 25% for dialysis centers (77% reduction), likely due to the home environment and less exposure to other people in the dialysis center and during transportation. One hundred twenty-six hospitalizations were reported during the study period, with only 12% related to dialysis (primarily due to volume overload and non-compliance with dialysis schedule and time).

**Figure 7.**
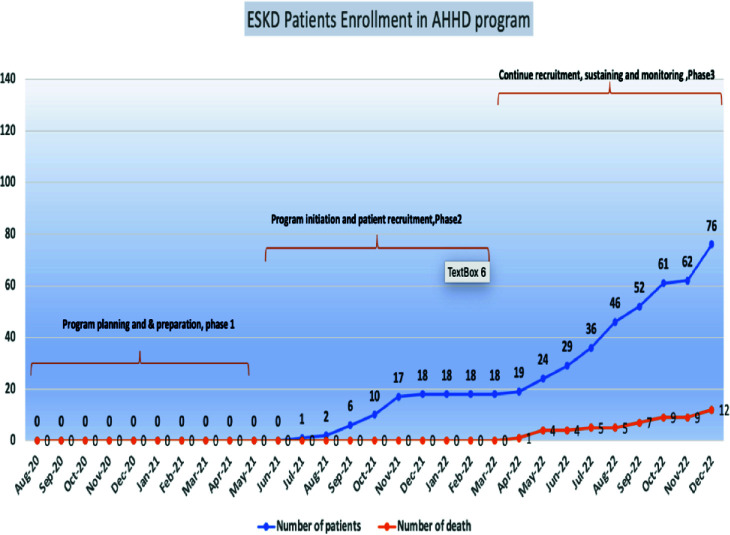
ESKD patient enrolled in AHHD program with reported mortality.

The AHHD program freed up crucial slots in hemodialysis centers, increasing availability by 10% as it transitioned eligible patients to home-based care. Our program overall was cost-effective and reduced costs by an estimated 25% (mostly related to savings in ambulance transfer costs), resulting in savings estimated at 1.4 million Qatari Riyals. Ambulance utilization for dialysis patient transport was reduced by 40%. Figure 7 summarizes the study recruitment and mortality. Patients reported an extraordinary satisfaction rate of 99%, with a retention rate of 97% overall. Patients who shifted to AHHD reported improved sleep, reduced stress, decreased fatigue, and increased participation in social activities. AHHD eliminated transportation challenges, traffic hassles, and waiting periods, which allowed patients to spend more time with loved ones and engage in social activities.

## Conclusion

The AHHD program is a superior, cost-effective, and clinically advantageous alternative to traditional in-center hemodialysis for targeted patients. It showed excellent patient safety, with no incidents of bleeding or falls and a reduced infection rate risk. The AHHD program aligns with HMC’s vision of person-centered care, emphasizing patient autonomy and superior care experiences.

## Conflict of Interest Statement

The authors declare no conflict of interest.

## Acknowledgments

M. Sheriff, A. Jadhav, A. Antony, N. Veneracion, S. Paulson, N. Mansoory, E. Hoe, S. Chembolu, B. Jacob, H. Hamdan, I. Khater, H. Al-Qahtani, M. Alali, R. Alnoor, E. Mohamed, A. Elnoor, M. Filali, HMC administration, and all dialysis leadership and team members for their valuable contribution to the success of the program and supporting this manuscript.

## Authors’ Contributions

AH, MYA, and ME: conceptualization, methodology, writing-original draft preparation, project administration, and formal analysis. AA, SC, HT, FY, RI, SJ, and TA: data curation, literature review, final approval of the version to be published, and agreement to be accountable for all aspects of the work in ensuring that questions related to the accuracy or integrity of any part of the work are appropriately investigated and resolved. MA, and HM: writing-review, visualization, and editing. All authors have read and agreed to the published version of the manuscript.

## Institutional Review Board Statement

The study was conducted in accordance with the Declaration of Helsinki and approved by the Institutional Review Board of the local ethics committee of the Medical Research Center at Hamad Medical Cooperation (protocol code: MRC-01-23-081, date: 20 February 2023).

## Informed Consent Statement

Patient consent was waived due to the retrospective, anonymous character of this study.

## Data Availability Statement

The original contributions presented in the study are included in the article, further inquiries can be directed to the corresponding author.

## Figures and Tables

**Table 1.** Summarizes challenges met in establishing our assisted home hemodialysis program.

**Challenge category**	**Details**
Recruiting and training	- Ensure that the hiring and training process details are specific and highlight any unique aspects or challenges faced in recruiting each role (nephrologists, nurses, vascular surgeons, biomedical engineers, dieticians, and clinical pharmacists).
Patient interaction	- Patient selection, program introduction, and multidisciplinary team meetings with patients and families.
	- The concept change from in-center hemodialysis to home hemodialysis was met with resistance and fear from patients and family members (HD away from the center, social aspects, space requirement (dedicated room), etc.).
	- Ensuring that patients’ homes meet safety standards through multiple home visits.
Documentation and reporting	- Providing access and training for new team members to electronic medical records (EMR) for proper documentation.
	- Activated laboratory orders, printed stickers, and generated accepted ICT encounters at the HHD setup.
	- Scanning and filing treatment reports in EMR.
	- Reviewed and audited various reports and addressed documentation errors.
Medical consumables and medications	- Communicating with the HMC pharmacy for medication access.
	- Obtaining high-risk medications to be used in the home setting (like tissue plasminogen activator (TPA) catheter locks, which took a year’s worth of efforts among our team, pharmacy, ensure safety, etc.) and ensuring their efficient and safe preparation and use.
	- Addressing challenges with non-formulary medications and providing narcotic prescriptions as needed.
	- Disposing of biohazard waste.
Technical challenges	- Setting up dialysis equipment at patients’ homes presented significant obstacles.
	- Interruptions in the water supply and high incoming water temperatures due to Qatar’s hot and humid climate, especially during the lengthy summer, posed further challenges. Solutions were implemented by installing a dedicated water tank and a portable chiller, ensuring a stable and constant water supply at an acceptable temperature for machine operations.
	- Testing water and dialysate samples for microbiology per our standards became challenging due to a substantial increase in the monthly sample numbers. A meticulous rescheduling and communication strategy was devised to manage proper sampling and testing for all home dialysis water samples monthly, ensuring consistency in meeting our quality standards.
Interdisciplinary coordination	- Coordinating with the emergency department for patient referral.
	- Addressing challenges related to communication with laboratory personnel.
	- Arranging meetings for HHD patients with home health care services.
Operational Processes	- Maintaining and verifying daily and monthly census data
	- Generating staff files based on HMC standards
	- Conduct staff rounds and ensure competency and compliance with HMC standards.
Communication and Patient	- Ensuring proper entry into patients’ homes with family permissions
Confidentiality	- Handling critical laboratory results for outsourcing and ensuring smooth communication.
Monitoring quality of care	- Creating and updating monthly key performance indicators (KPIs).
	- Monitor KPIs on a regular frequency with an action plan.
Covid-19 pandemic	- Handling all challenges related to patients’ isolation and social distancing.
	- Handling delays in equipment and consumable delivery.

**Table:** Table of abbreviations.

**Abbreviations**	**Term**
AHHD	Assisted Home Hemodialysis
ESKD	End-stage kidney disease
HD	In-center hemodialysis
HHD	Home HD
PD	Peritoneal dialysis
COVID-19	Coronavirus Disease 2019
